# A new and spontaneous animal model for ankylosing spondylitis is found in cynomolgus monkeys

**DOI:** 10.1186/s13075-021-02679-5

**Published:** 2022-01-03

**Authors:** Huanhuan Jia, Meili Chen, Yanzhen Cai, Xiaoling Luo, Gang Hou, Yongfeng Li, Chunmei Cai, Jun Chen, Qingnan Li, Kai-Kei Miu, Sin-Hang Fung, Zhangting Wang, Ren Huang, Huiyong Shen, Li Lu

**Affiliations:** 1 School of Life Science and Biopharmacy, Guangdong Provincial Key Laboratory of Pharmaceutical Bioactive Substances, Guangdong Pharmaceutical University, Guangzhou, China; 2 Guangdong Key Laboratory of Laboratory Animals, Guangdong Laboratory Animals Monitoring Institute, Guangzhou, China; 3 Department of Orthopedics, The Third Affiliated Hospital of Sun Yat-Sen University, Guangzhou, China; 4 Guangzhou Blooming-Spring Biological Research Institute, Guangzhou, China; 5 Developmental and Regenerative Biology Theme, School of Biomedical Sciences, Faculty of Medicine, The Chinese University of Hong Kong, Shatin, Hong Kong SAR China; 6 Department of Orthopedics, The Eighth Affiliated Hospital of Sun Yat-sen University, Shenzhen, China

**Keywords:** Ankylosing spondylitis, Cynomolgus monkeys, Animal model, Spontaneous, Hematological testing, Radiographic examination, Family aggregation analysis, Pathological analysis

## Abstract

**Background:**

Ankylosing spondylitis is a progressive, disabling joint disease that affects millions worldwide. Given its unclear etiology, studies of ankylosing spondylitis relied heavily on drug-induced or transgenic rodent models which retain only partial clinical features. There is obviously a lack of a useful disease model to conduct comprehensive mechanistic studies.

**Methods:**

We followed a group of cynomolgus monkeys having joint lesions reported of spinal stiffness for 2 years by conducting hematological testing, radiographic examination, family aggregation analysis, pathological analysis, and genetic testing.

**Results:**

The results confirmed that these diseased animals suffered from spontaneous ankylosing spondylitis with clinical features recapitulating human ankylosing spondylitis disease progression, manifested by pathological changes and biochemical indicators similar to that of ankylosing spondylitis patients.

**Conclusion:**

The study offers a promising non-human primate model for spontaneous ankylosing spondylitis which may serve as an excellent substitute for its pre-clinical research.

**Supplementary Information:**

The online version contains supplementary material available at 10.1186/s13075-021-02679-5.

## Background

Ankylosing spondylitis (AS) is a progressively disabling disease that affects both axial and peripheral joints. Clinically, a patient with AS would be described as suffering an inflammatory pain in the spine surmounting to immobility. AS targets young males aged from 15 to 30 years old than that of female individuals, with an odds ratio of roughly 2:1, while it appears rare in those aging more than 40 [1]. The incidence rate of AS in the Chinese population is about 0.24% [2]. Nonetheless, after years of active clinical research, there are few pathological descriptions [3] which largely inform associated genetic and environmental risks [4–6]. The missing etiology of AS accounted for the bottleneck of drug development.

In clinical settings, diagnosis and staging of AS relied on limited longitudinal imaging analysis (MRI and X-ray) with inadequate sensitivity. Collecting relevant biopsy samples from spinal lesions was again incomplete as limited by the sample size of matching axial anatomical locations indicative of bone erosion and syndesmophyte [7–9]. There is also a limited representation of AS in advanced disease stages already undergone osteoproliferative transitions. It is therefore necessary to look for experimentally feasible alternative subjects which spontaneously develop clinical features resembling that of AS patients as a model for research.

Yet, it remains difficult to benchmark and produce disease animal models with high similarity to clinical AS patients without knowing the etiology. Lab rodents including HLA-B27 humanized transgenic mouse model [10], ERAP1-deficient mouse model [11], or inflammation-inducing mouse models [12] only recapitulated the genetic susceptibility aspect of AS partially while often borne with inconsistent pathology.

Significant efforts in screening more than 20,000 cynomolgus monkeys within the Guangdong Province for spinal deformity and immobility between the ages of 4 and 12 years old allowed pilot X-ray examination in these monkeys bearing features resembling AS patients. A subsequent 2-year follow-up spinal and hematological examination in these monkeys included imaging (X-ray, MRI, and CT), family recurrence analysis, genetic testing, and pathological analysis with strikingly consistent pathological changes as in AS patients. Our report suggests cynomolgus monkeys with spinal deformity as an excellent surrogate primate model of spontaneous AS.

## Methods

### Animals

All animals for the initial screen are adopted from Guangdong Chunsheng Biotechnology Development Co., Ltd., license number: SCXK (Guangdong) 2014-0027. The animals were housed under room temperature between 24 and 30°C with a relative humidity fluctuating around 60%, operating under at 12h/12h normal light/dark cycle illumination.

A universal goniometer was used to evaluate the curvature of spine and the range of motion (ROM) of both knee joint and hip joint for the screen under standard procedures described in details elsewhere [13].

### X-ray examination and CT scan

We performed X-ray examination on monkeys with mobility problems aged 4–12 years using HF400VA X-ray system (Mikasa, Japan) every 6 months within 2 years. The radiographic score were evaluated by two independent trained readers by the modified Stoke Ankylosing Spondylitis Spinal Score (mSASSS) [14]. A 0.5-mm-thick of CT section images was obtained by an Aquilion 64 scanner (Toshiba, Japan) with tube current and potential set at 250 mA and 135 kV, respectively.

### Hematological examination and cytokine detection

Blood samples prepared along clinical assessment were kept frozen at −80°C until hematological examination were performed by the biochemical analyzer (HITACHI, 3100, Japan). Tumor necrosis factor α (TNF-α), vascular endothelial growth factor (VEGF), C-reactive protein (CRP), an anti-cyclic peptide containing citrulline antibody (anti-CCP antibody), anti-Streptolysin O (ASO), Procalcitonin (PCT), and rheumatoid factors ( IgG-RF & IgM-RF) levels were measured with monkey-specific ELISA kits, respectively ( Supplementary Table 1).

### Paraffin section and staining

The lumbar vertebra and caudal were decalcified with EDTA, followed by dehydration and paraffin embedding. Sections were stained with Mayer’s hematoxylin and eosin (H&E) or 0.1% safranin-O/0.05% fast green (Sigma-Aldrich).

### SNP genotyping

Genomic DNA was extracted from the peripheral blood with Whole Blood Genomic DNA Extraction Kit (Takara) before genotyping of SNPs determined by the SNaPshot Assay (Shanghai General BioTech Co., Ltd.) with primer pairs shown in Supplementary Table 2. The SNPshot sequencing relied on monkey-specific but not degenerate primer pair spanning the homologous site with OR predicted to be relevant to AS adapted from the reference genome in NCBI annotate 101 for the species. As for the WGS sequencing, raw reads were first trimmed by Cutadapt (v3.4). Then, the adapter-trimmed reads were aligned to Burrows–Wheeler aligner (v0.7.17). PCR duplicates were removed using Picard MarkDuplicates. Variants calling were performed by GATK (v 4.2.1) germline short variant discovery pipeline and following the GATK best practices. The pipeline utilizes HaplotypeCaller and filtered by GATK CNNScoreVariants and FilterVariantTrances. Variants are further filtered with “AC=2” for homozygous alternate alleles and “AC=1” for heterozygous alternate alleles. Mutational variants were functionally annotated using SnpSift (v.4.3.1).

### Statistics

All values are presented as mean ± SD analyzed with SPSS (Version 20.0) for Windows. The normality of distributions was tested using the Kolmogorov-Smirnov test. Comparisons of normally distributed study parameters are performed using one-way ANOVA. *P* values < 0.05 were considered statistically significant.

## Results

### Physical examination

**Fig. 1:**
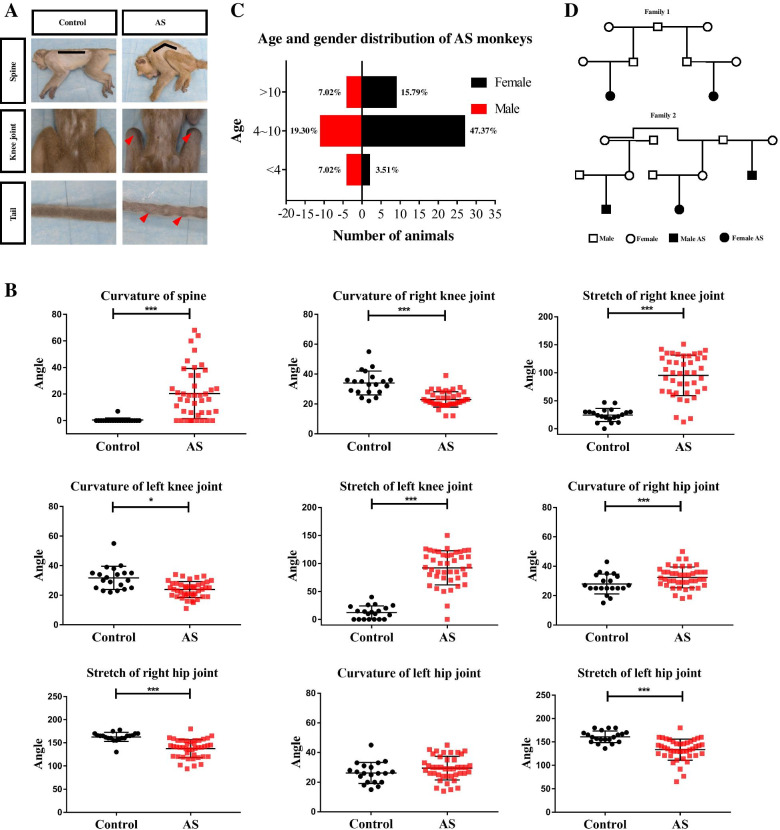
Physical examination and epidemiology investigation of Cynomolgus monkeys. **A** Comparison of surface observations between normal and diseased animals. Representative images of cynomolgus monkey’s spines, knees, and tails. The spines of the diseased animal are curved (black line), the knee joints are swollen, and the tail appears as nodular (red arrowhead). **B** Joint motilities were evaluated by a measuring ruler as descried in the methods. Values on graphs are presented mean ± SD. *p* values calculated by Student’s *t* test, where *p < 0.05, ***p < 0.001, compared with control (Control: *n*=20, AS: *n*=42). **C** Age and gender distribution of AS monkeys. The age of the animals counted in the figure is the age at which the animals were diagnosed (*n*=57). **D** Family trees of 2 monkey families. Squares = males; circles = females; darkened squares and circles = monkeys with ankylosing spondylitis

The cynomolgus monkeys with joint abnormalities have symptoms either as a hunchback, muscle atrophy, lameness, tail stiffness, restricted spinal activity, or spinal rigidity. Both the spine and peripheral joints of these monkeys were swollen and highly immobile (Fig. 1A). Most of them have enlarged, stiff knees with limited mobility and stretch together with stiff tail and bamboo-like exterior.

Aside from postures and movements, we also measured joint mobility. The curvatures of the spine of diseased monkeys are significantly greater than normal (Fig. 1B). While the curvatures of bilateral knee joints were significantly reduced with increased stretch, that in the right hip joint was significantly increased. Finally, the bilateral hip extensions were also significantly reduced but none were found in the bilateral elbow joints (Supplementary Table 3 and 4).

### Incidence and family aggregation

Through daily monitoring and cross-checking by the farm veterinarian, about 200 animals with mobility problems were identified out of the whole farm comprised of 20,000 monkeys in total. The refined identification allowed 57 cynomolgus monkeys to be diagnosed with AS, and the incidence rate was about 0.275% similar to the likelihood as in humans. The age and gender distribution of these AS cynomolgus monkeys were correspondingly tabulated (Fig. 1C). With that, we further checked animal archives for husbandry information just so to confirm diseased animals were close relatives (Fig. 1D). It was found that 5 AS cynomolgus monkeys (5/57, 8.77%) had family aggregation distributed in 2 separated bloodlines.

### X-ray examination and CT scan

**Fig. 2:**
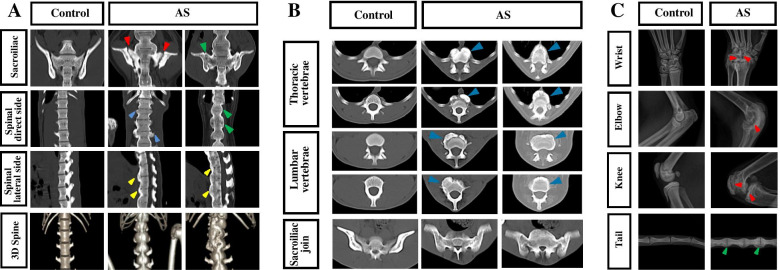
CT scan and X-ray examinations of axial and peripheral joints in Cynomolgus monkeys. **A** Representative longitudinal section CT-scan images of the sacroiliac joint and spine (L1-S1) of Cynomolgus monkeys. The AS monkeys showing extensive bone erosion (red arrowheads), syndesmophyte formation (blue arrowheads), and joint fusion (green arrowheads) in the sacroiliac joint and spine. The bony spurs (yellow arrowheads) and vertebral bodies fuse together, the spine appears as a single piece and is described as the bamboo spine (3D spine). **B** Representative transverse section CT-scan images of thoracic vertebrae, lumbar vertebrae, and sacroiliac joint of Cynomolgus monkeys. The AS monkeys showing extensive syndesmophyte formation (blue arrowheads) in the lumbar and thoracic vertebrae, with joint fusion in the sacroiliac joints. **C** X-ray examinations of the peripheral joints in Cynomolgus monkeys. Representative X-ray images of the wrists, elbows, knees, and tails of Cynomolgus monkeys, showing extensive bone erosion and/or syndesmophyte formation (red arrowheads) and joint fusion (green arrowheads) in the AS monkeys

X-ray examination showed that 57 cynomolgus monkeys had variant joint abnormalities in the central joint, tail joint, and limb joints (Table 1). Radiographic results reveal erosive changes at the corners of the vertebral bodies quite early with the outgrowth of syndesmophytes at later stages. When these syndesmophytes fuse with adjacent vertebral bodies, the apparent single bone piece is aptly described as bamboo spine (Fig. 2A). CT scan had indicated a general rough surface for sacroiliac joints fused in some severe cases (Fig. 2B). Similar lesions can also be found for the knee joints, wrist joints, and at the vertebral of the tails (Fig. 2C).

**Table 1:** Statistics of lesion involvement sites in AS cynomolgus monkeys

Involvement site	Before AS (*n*=46)	After AS (*n*=57)
Axial joint		
Sacroiliac joint	29 (63.0)	57 (100.0)
Caudal vertebrae	26 (56.5)	48 (84.2)
Lumbar vertebra	23 (50.0)	53 (93.0)
Thoracic vertebra	18 (39.1)	36 (63.2)
Cervical vertebra	13 (28.3)	24 (42.1)
Peripheral joint		
Knee joint	41 (89.1)	55 (96.5)
Elbow joint	3 (6.5)	8 (14.0)
Wrist joint	6 (13.0)	6 (10.5)
Ankle joint	1 (2.2)	6 (10.5)
Toe joint	0	3 (5.3)
Finger joint	0	3 (5.3)

Note: Data presented as *n* (%).

### The radiographic progressing in AS cynomolgus monkeys

**Fig. 3:**
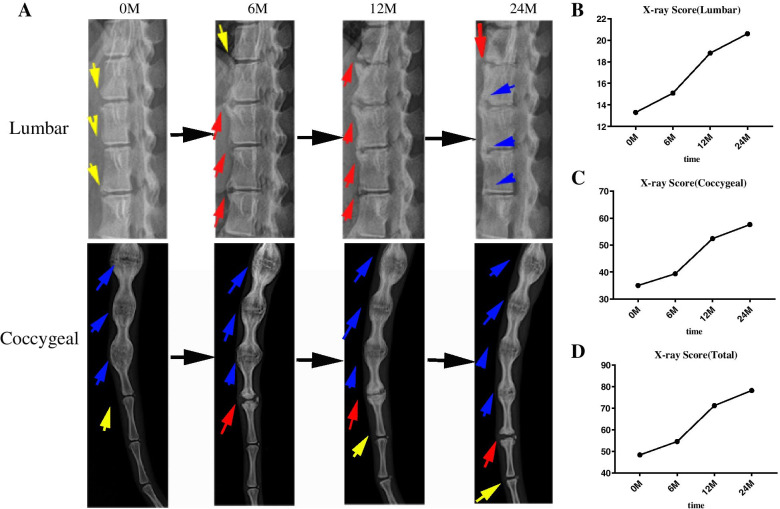
Evaluation of the radiographic progression of AS cynomolgus in two years. **A** Progression of X-rays in the lumbar and coccygeal vertebra of AS cynomolgus within 2 years (yellow arrow points to the erosion and/or sclerosis and/or squaring; red arrow points to syndesmophyte (non-bridging); blue arrow points to total bony bridging between upper and lower vertebral edges). **B** Broken line diagram of the lumbar score. **C** Broken line diagram of the coccygeal score. **D** Broken line diagram of total score(*n*=10)

To characterize the disease progression in AS monkeys, we performed X-ray examinations of disease animals once every 6 months within 2 years. At the time of the first X-ray examination, the animals were within the age of 4–8 years. We found a progressive disease consistent with AS patients, firstly bone loss followed by bone spurs bridging vertebrae (Fig. 3A). The X-ray time series aided in calculating the required time for AS monkeys to shift from bone erosion to spur formation (Fig. 3B–D).

### Cytokines of inflammation

**Fig. 4:**
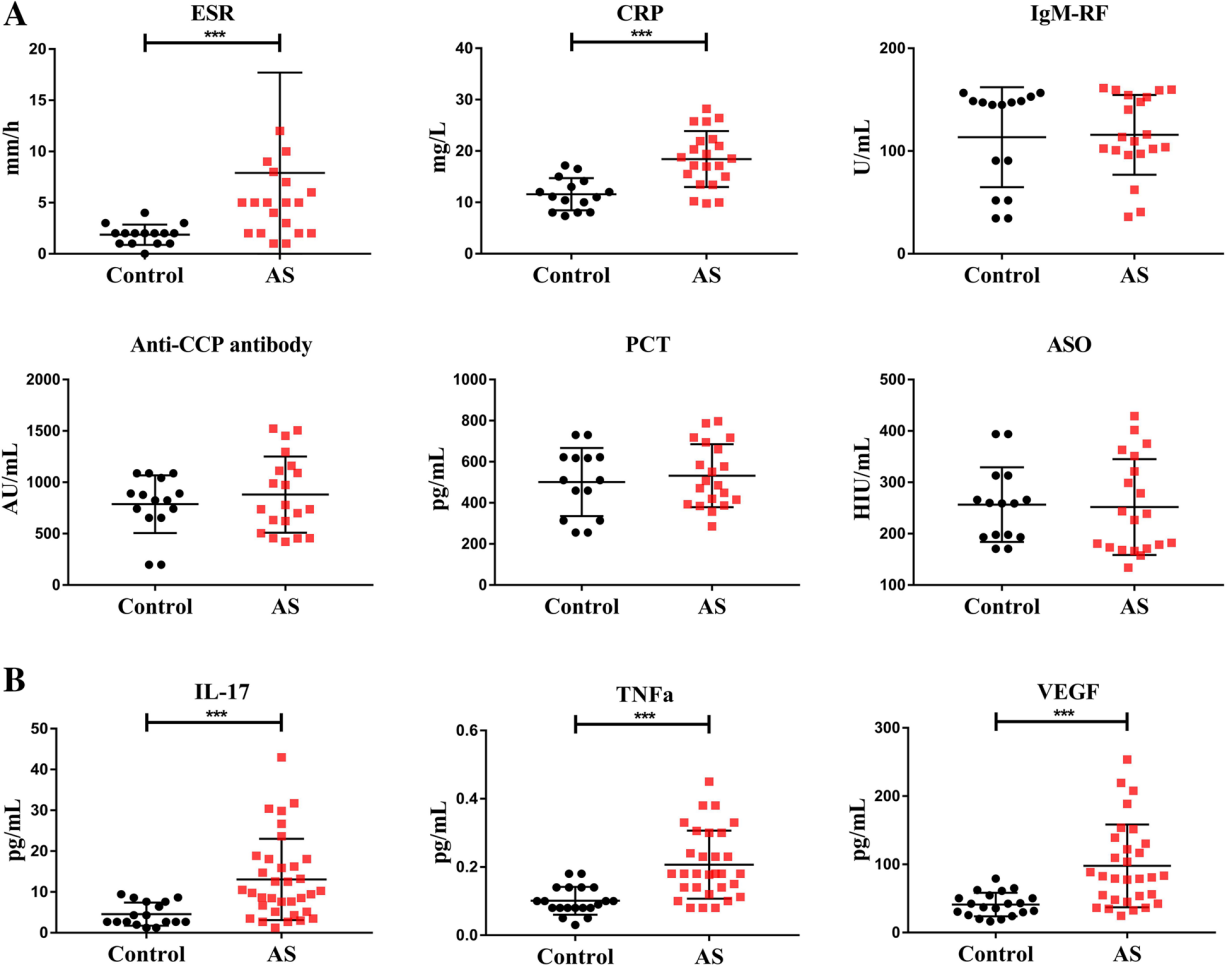
The hematological parameters and inflammatory cytokines of cynomolgus monkeys. **A** Evaluation of inflammatory cytokines of cynomolgus monkeys (control: *n*=15, AS: *n*=20). The features of inflammatory cytokines include ESR (erythrocyte sedimentation rate), CRP (C-reactive protein), IgM-RF (immunoglobulin M-rheumatoid factor), anti-CCP antibody (anti-cyclic peptide containing citrulline antibody), PCT (procalcitonin), and ASO (anti-Streptolysin O). **B** Comparison of blood cell classification between normal and diseased cynomolgus monkeys (control: *n*=17, AS: *n*=34). The characteristic change of inflammatory cells is described by IL-17 (interleukin 17), TNF-α (tumor necrosis factor α), and VEGF (vascular endothelial growth factor). Values on graphs are the mean ± SD. *p* values calculated by Student’s *t* test, where *p < 0.05, ***p < 0.001, compared with control

To further distinguish AS from other similar diseases, we checked the expression of serum cytokines in diseased animals (Fig. 4A). When compared to normal monkeys, AS monkeys showed a significant increase in ESR and CRP levels, which represents two important indicators of inflammatory activity. However, there was no significant change in IgM-RF, anti-CCP antibody, PCT, and ASO levels. The serum cytokines examination revealed that IL-17, VEGF, and TNF-α levels were significantly elevated in AS monkeys when compared to normal monkeys (Fig. 4B), while IL-1β, IL-2, IL-8, IL-6, IL-10, IL-12, and IFN-ɣ levels did not deviate significantly (data not shown here). Importantly, IL-17 [15, 16], VEGF [17, 18], and TNF-α [19] elevation are deemed characteristic features of AS clinical patients. The above results suggest that the diseased animals demonstrated similar characteristics as in the AS patients in clinical settings.

### Hematological examination

**Fig. 5:**
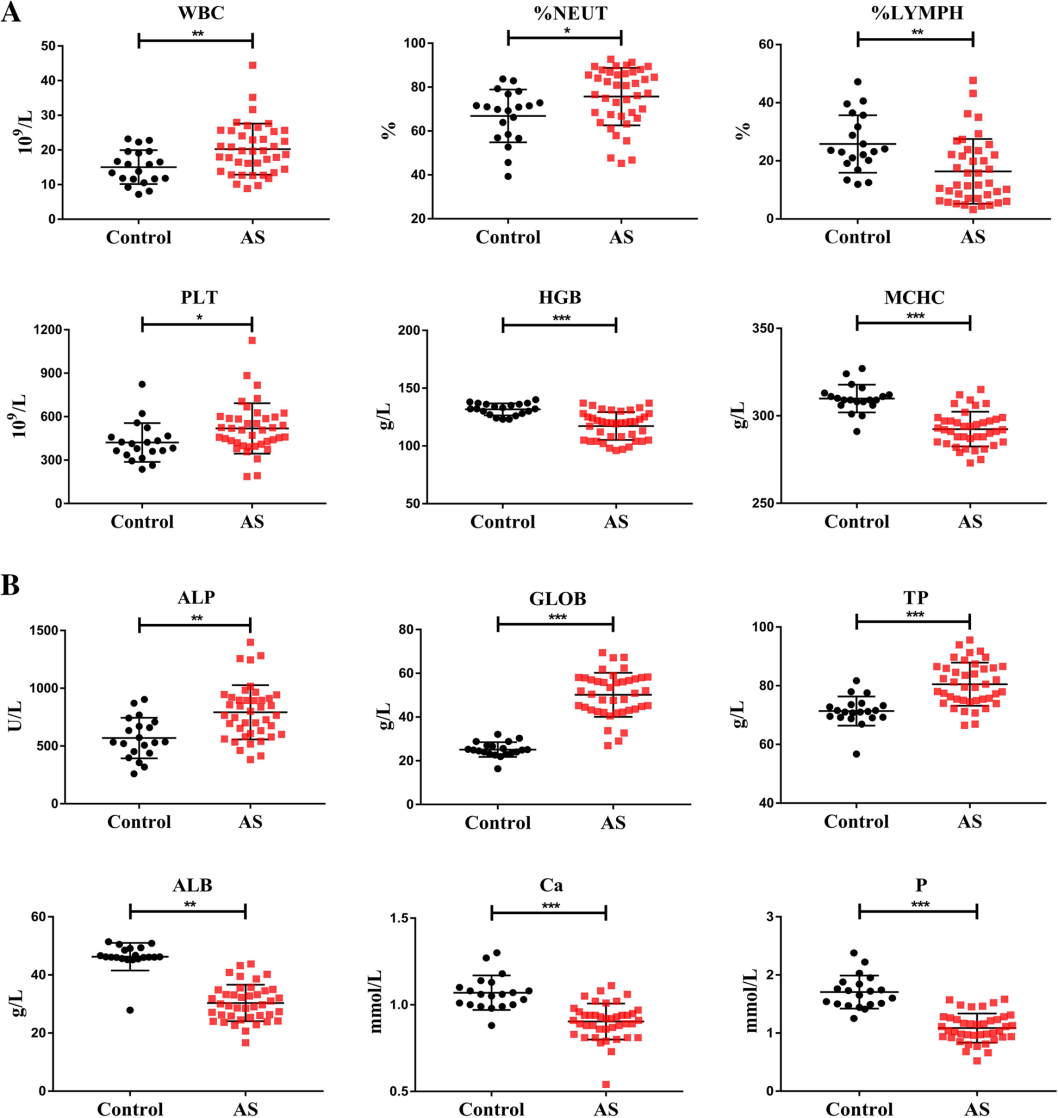
Comparison of serum biochemistry between normal and diseased cynomolgus monkeys. **A** Inflammatory reaction is characterized as the change of WBC (white blood cell), %NEUT (percentage of neutrophils), %LYMPH (percentage of lymphocyte), PLT (blood platelet), HGB (hemoglobin), and MCHC (mean corpuscular hemoglobin concentration) of diseased cynomolgus monkeys. **B** Serum ALP (alkaline phosphatase), GLOB (globulin), TP (total protein), ALB (albumin), Ca (calcium), and P (phosphorus) of normal and diseased cynomolgus monkeys. Values on graphs are the mean ± SD. *p* values calculated by Student’s *t* test, where *p < 0.05, **p < 0.01, ***p < 0.001, compared with control (control: *n*=20, AS: *n*=41)

Hematological examination showed that PLT increased significantly, while HGB and MCHC decreased in AS monkeys when compared to control as an indication of mild anemia (Fig. 5). WBC increased significantly suggested these AS monkeys suffer from inflammatory responses. Subsequent cytology analysis revealed that neither leukocytes, eosinophils, basophils, nor monocytes were significantly expanded in population, but lymphocytes were indeed decreased while neutrophils were increased significantly in the AS group contributing to the higher WBC count. Serum biochemical indicators including ALP, GLOB, and TP increased significantly in AS likely out of nutritional malabsorption and inflammation, while serum ALB, Ca, and P decreased significantly indicative of bone metabolic changes.

### Pathological changes of the lumbar vertebrae in AS animals

In AS cynomolgus lumbar vertebral sections, we observed cartilage destruction, chondroid metaplasia, bony spur formation, synovial hyperplasia, intra-articular fibrous strands, and vascular proliferation just as that observed in clinical samples. This is in stark contrast to healthy monkeys where intervertebral annulus fibrosus were arranged neatly while chondrocytes were arranged in longitudinal rows within the cartilage plate connecting the subchondral bone. The intervertebral disc space of the AS cynomolgus vertebral body gradually became narrower as the disease progressed until the final disappearance when the cartilage is fused.

**Fig. 6:**
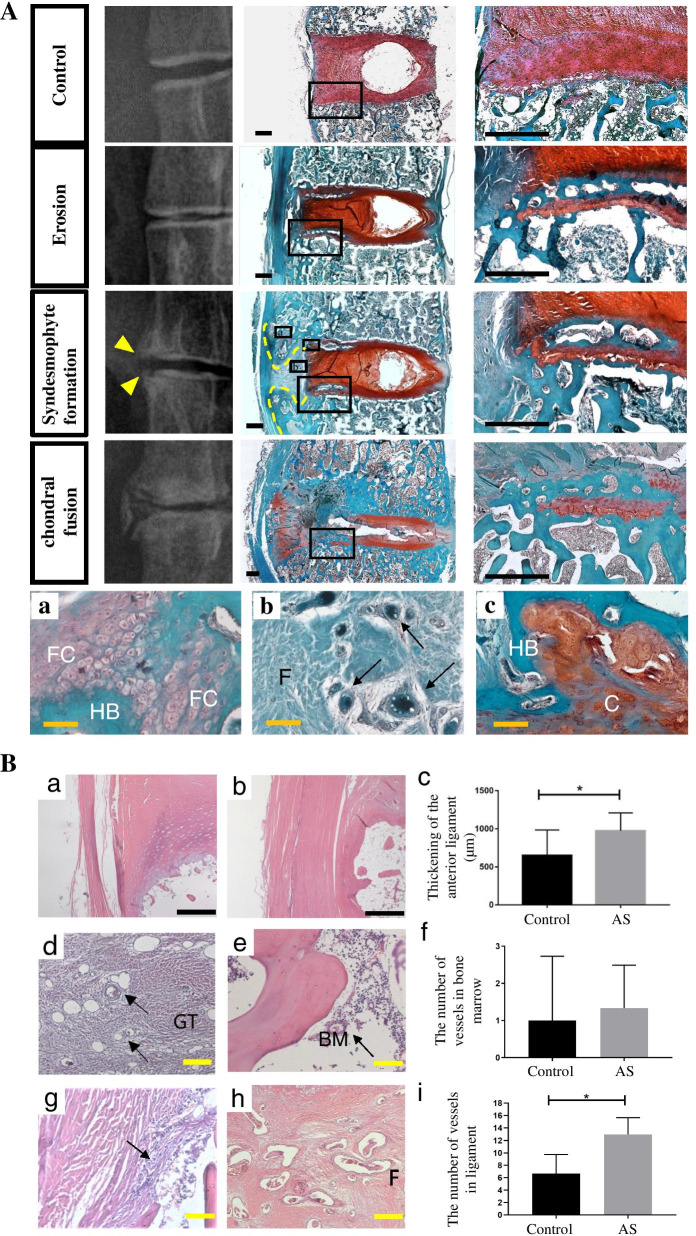
Pathological changes of lumbar vertebrae. **A** Histopathologic evaluation of the ankylosed intervertebral joints. The spines from 6- to 10-year-old normal and diseased animals (*n* = 3) were harvested, fixed, and stained. The bone is stained blue while the cartilage, and annulus fibrosus are stained red. Representative safranin-O/fast green images of normal animals’ joint demonstrating normal joint cavity, healthy cartilage, and annulus fibrosus. Representative safranin-O/fast green images of diseased animals intervertebral joints demonstrating cartilage disruption of the disc by a heterotopic ossification tissue (magnification of the boxed area in erosion vertebrae showed bony invasion and cartilage damage). Heterotopic bone formation was shown at the edge of the vertebral bodies. The area inside the yellow dotted line was syndesmophytes, which corresponds to the place pointed by the yellow arrow on the X-ray image. The intervertebral disc of the AS cynomolgus became narrower as the disease progress until the final disappearance when cartilage fused. Representative safranin-O/fast green of diseased animals syndesmophyte demonstrating heterotopic bone, fibrocartilage (**a**), osteoclasts (**b** black arrows) and cartilage calcifications (**c**). FC fibrocartilage, F fibrous tissue, HB heterotopic Bone, C cartilage plate. Black scale bar = 1mm. Yellow scale bar = 100μm. **B a** Representative hematoxylin-eosin (HE) staining images of normal animals joint demonstrating normal anterior ligament. **b** Representative HE staining images of AS animals joint demonstrating anterior ligament. **c** Thickening of the anterior ligament. **d** Representative HE staining images of AS animals joint demonstrating granulation tissue (GT). There were a large amount of small blood vessels in this GT. **e** An amount of small blood vessels in the bone marrow (BM). **f** The number of vessels in the bone marrow. **g** An amount of inflammatory cell infiltration in the articular capsule. **h** There were an amount of small blood vessels in fibrous tissue (F) of the ligament. **i** The number of vessels in the ligament. *p* values calculated by Student’s *t* test, where *p < 0.05, compared with control (control: *n*=7, AS: *n*=12). Black scale bar = 1mm. Yellow scale bar = 100μm.

In the early stage, thickening of the anterior ligament and synovial hyperplasia could be observed, with inflammatory cell infiltration and obvious fibrous tissue hyperplasia (Fig. 6A). More importantly, there were a large amount of small blood vessels and osteoclasts in these fibrous tissues (Fig. 6Ab and Ac). Bony nodules could be observed in the deep zone of the cartilage plate near the subchondral plate (Fig. 6A).

The spur formation area was frequently situated around the anterior vertebral ligament during the syndesmophyte formation stage, where they extended from the vertebral body edge of the upper and lower sides of the disc, surrounding the proliferating fibrous tissue. Inside the spur formation area, heterotopic ossification and chondroid metaplastic foci could be found (Fig. 6Aa). Cells inside the chondroid metaplastic foci, also known as calcified fibrocartilage, were not densely packed while also rarely arranged in columns or hypertrophy unlike that in normal cartilage plates. Finally, both the original and the ectopic cartilaginous tissues were replaced by bone at the cartilage fusion stage. Here, the original cartilages were fused, and chondral fusion was the predominant mode of ankyloses (Fig. 6A).

Through H&E staining, we found a significant thickening of the anterior ligaments in the AS animals joint (Fig. 6Bb and Bc), and large numbers of the small blood vessels in the bone marrow and fibrous tissue of the ligament (Fig. 6Be and Bh). More importantly, there is a large amount of inflammatory cell infiltration in the articular capsule, indicating that enthesitis is a major feature of AS (Fig. 6Bg).

### Identification of disease-relevant genetic traits

The MHC genotype is a key determinant for medical research involving immune and inflammatory responses while HLA-B27 encodes a typical human MHC Class B allele pronounced as the major AS-susceptibility gene. Notably, several top hits for the non-coding SNP database had also spanned around the human MHC-B locus. As such, we focused on this highly complex locus for its likely strong association with AS phenotype also in cynomolgus.

**Fig. 7:**
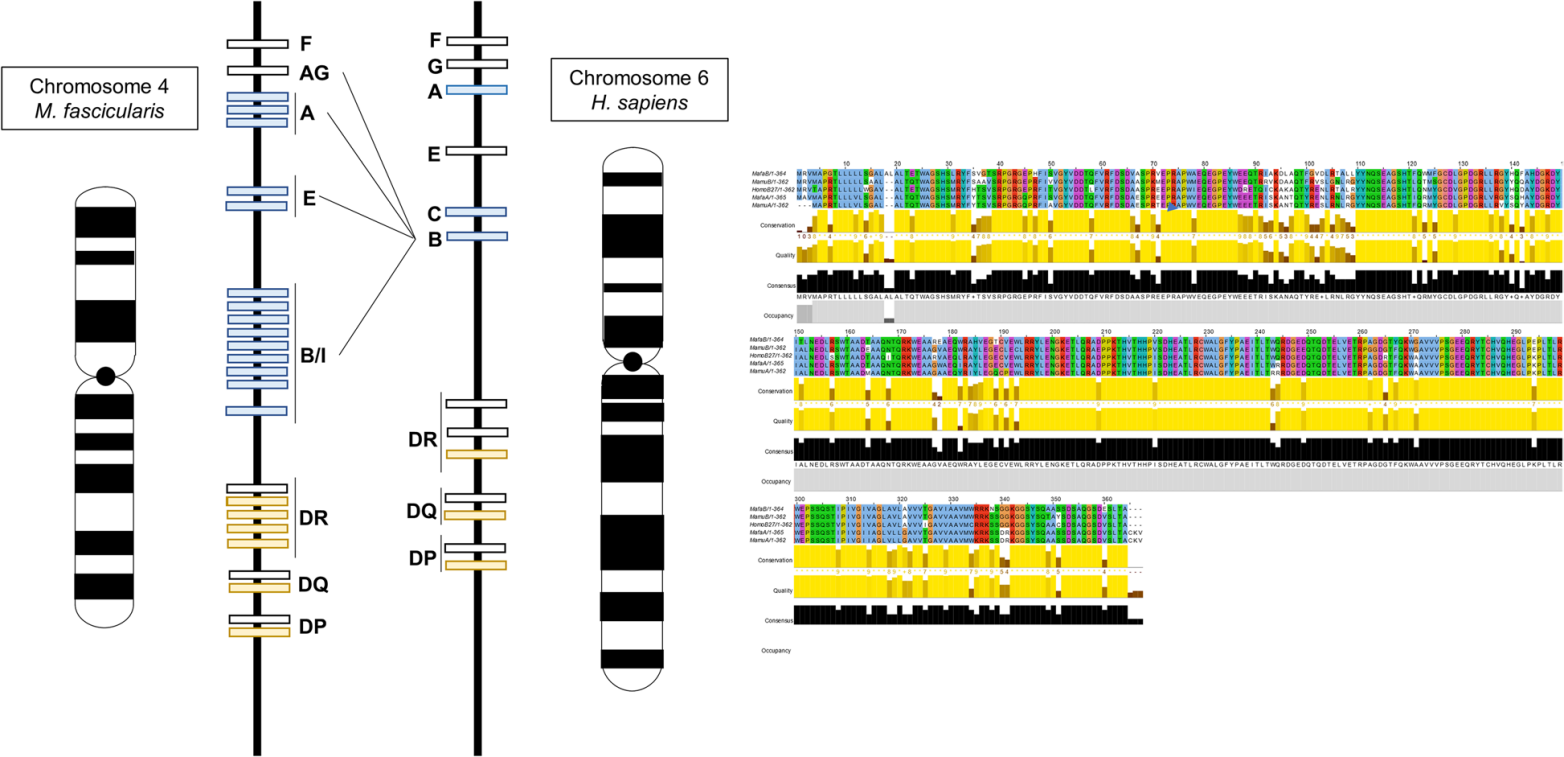
Comparative genome maps of representative MHC genes in humans and cynomolgus macaques. HLA and Mafa mean human and cynomolgus macaque MHCs, respectively. Orange boxes indicate classical class I genes, HLA-A, HLA-B, HLA-C, and their Mafa orthologs (Mafa-A, Mafa-B, and Mafa-I), in the class I sub-region, and blue boxes indicate classical class II genes, HLA-DR, HLA-DQ, HLA-DP, and their Mafa orthologs (Mafa-DR, Mafa-DQ, and Mafa-DP), in the class II sub-region. Numbers under boxes and in parentheses indicate allele numbers reported by the IPD-IMGT/HLA database release 3.43.0 in January 2021 in humans (Available from: https://www.ebi.ac.uk/ipd/imgt/hla/) and the IPD-MHC database release 3.0.0.1 in February 2018 in cynomolgus macaques (Available from:https://www.ebi.ac.uk/ ipd/mhc/group/NHP/allele/Mafa).

Interestingly, we understand that macaques evolved without any copy of Mhc-C but Mhc-A and Mhc-B genes normally exist more than one copy per haplotype, while their functional redundancy prevented us from making a judgment in naming only alleles from the Mafa-B cluster as likely candidates for HLA-B27-like allele. As a precaution, we sought to identify sequences with high homology with HLA-B reference loci at the genomic level which returned Mafa-A alleles with a predicted higher sequence proximity to HLA-B than Mafa-B alleles (Fig. 7, Table 2). Next, we performed BlastP comparison for all available Mafa-A & Mafa-B IPD-MHC allele database with the commonest AS MHC allele HLA-B2705 which revealed Mafa-A1*027:02, Mafa-A1*007:04, and Mafa-A2*05:48 alleles as top-hits (Table 3).

**Table 2:** HLA comparison in genomic levels

*H. sapiens*	*M. fascicularis*	
Gene symbol	Gene symbol	Gene synonym
HLA-B	HFE	Homeostatic iron regulator
HLA-B	LOC102114865	MAFA-A, MAFA-A1, MAFA-AG, MAFA-AG2, MAFA-AG3, HLA class I histocompatibility antigen, A-11 alpha chain-like
HLA-B	LOC102116897	MAFA, MAFA-B, MAFA-I, HLA class I histocompatibility antigen, B-7 alpha chain
HLA-B	LOC102117143	MAFA-A, MAFA-A1, MAFA-A2, MAFA-A3, HLA class I histocompatibility antigen, A-25 alpha chain-like
HLA-B	LOC102120132	MAFA, MAFA-B, MAFA-I, HLA class I histocompatibility antigen B-27 alpha chain-like
HLA-B	LOC102121775	MAFA, MAFA-B, class I histocompatibility antigen, Gogo-B*0103 alpha chain-like
HLA-B	LOC102123548	MAFA-B, MAFA-B17, HLA class I histocompatibility antigen, B-14 alpha chain-like
HLA-B	LOC102124611	Uncharacterized LOC102124611
HLA-B	LOC102125623	MAFA, MAFA-A, MAFA-A3, MAFA-A4, patr class I histocompatibility antigen, A-126 alpha chain
HLA-B	LOC102127128	Uncharacterized LOC102127128
HLA-B	LOC102132408	MAFA, MAFA-B, MAFA-B7, HLA class I histocompatibility antigen, B-14 alpha chain-like
HLA-B	LOC102132794	MAFA-A, MAFA-AG, HLA class I histocompatibility antigen, A-11 alpha chain-like
HLA-B	LOC102137111	Uncharacterized LOC102137111
HLA-B	LOC102138874	Popy class I histocompatibility antigen, alpha chain E, MAFA-E, MAFA-E-01, MAFA-E-02, MAFA-IE, MAFA-E1
HLA-B	LOC102138996	HLA class I histocompatibility antigen, B-49 alpha chain-like, MAFA-B, MAFA-B14
HLA-B	LOC102140389	MAFA-B, patr class I histocompatibility antigen, B-1 alpha chain-like
HLA-B	LOC102141578	Histone H3.1-like
HLA-B	LOC102141835	MAFA-B, HLA class I histocompatibility antigen, B-7 alpha chain-like
HLA-B	LOC102142617	MAFA-B, HLA class I histocompatibility antigen, B-14 alpha chain-like
HLA-B	LOC102143405	MAFA-B, patr class I histocompatibility antigen, A-5 alpha chain-like
HLA-B	LOC102143904	Uncharacterized LOC102143904
HLA-B	LOC102145054	HLA class I histocompatibility antigen, B-37 alpha chain-like

**Table 3:** B2705 allele comparison

Mafa loci	HLA B2705 loci	
Mafa-A1*007:04	B*27:05:06 B*27:05:08 B*27:05:09 B*27:05:11 B*27:05:12 B*27:05:13 B*27:05:14 B*27:05:15 B*27:05:16 B*27:05:17 B*27:05:19	B*27:05:20 B*27:05:21 B*27:05:22 B*27:05:24 B*27:05:25 B*27:05:26 B*27:05:27 B*27:05:28 B*27:05:29 B*27:05:30
Mafa-A1*027:02	B*27:05:02:01 B*27:05:02:02 B*27:05:02:03 B*27:05:02:04Q B*27:05:02:05 B*27:05:02:06 B*27:05:02:07 B*27:05:02:08 B*27:05:02:09 B*27:05:02:10 B*27:05:02:11 B*27:05:02:12 B*27:05:02:13 B*27:05:02:14 B*27:05:02:15 B*27:05:02:16 B*27:05:02:17 B*27:05:02:18 B*27:05:02:19 B*27:05:02:20 B*27:05:02:21 B*27:05:02:22 B*27:05:03 B*27:05:05 B*27:05:07 B*27:05:10	B*27:05:18:01 B*27:05:18:02 B*27:05:23 B*27:05:31 B*27:05:32 B*27:05:33 B*27:05:34 B*27:05:35 B*27:05:36 B*27:05:37 B*27:05:38 B*27:05:39 B*27:05:40 B*27:05:41 B*27:05:42 B*27:05:43 B*27:05:44 B*27:05:45 B*27:05:46 B*27:05:47 B*27:05:48 B*27:05:49 B*27:05:50 B*27:05:51 B*27:05:52 B*27:05:53
Mafa-A2*05:48	B*27:05:04	

In fact, all these functional annotated HLA-B27 homologs do not lie in the Mhc-B region but that is acceptable as expected, for it was already foreseen in its close relative Rhesus macaque (Macaca mulatta) where the peptide-binding specificity of alleles regardless of belonging to the A or B gene cluster, i.e., Mamu-A2*01:02, Mamu-B*010:01, and Mamu-B*003:01 were those stipulated as top-hits functional analog among all MHC alleles to the HLA-B27 supertype family [20].

In order to look for other relevant disease predictor locus for the group of AS monkeys, we performed genetic analysis for the 20 AS-related SNPs that have been reported in the literature [21–23], with a focus on particularly those reported to correlate to the HLA-B27 pathogenic pathway. In fact, the reference gene sequences in both human and cynomolgus monkeys retained the same nucleotide usage in 10 such SNP loci (rs30187, rs11209026, rs1004819, rs11465804, rs10889677, rs1495965, rs6556416, rs2297909, rs11249215, rs11616188); therefore, the discovery of any SNP change may indicate a high possibility of inheriting AS-relevant traits.

Unfortunately, paternal parenthood in these promiscuous cynomolgus monkeys was proven difficult and therefore a trio analysis to study the haplotype of MHC locus was virtually impossible. Relentlessly, we managed to follow through and produce whole-genome sequencing in a pair of healthy mother and disease son. With that, we checked carefully in all 10 disease-relevant SNP loci, including two homolog SNP with the highest OR for AS, rs4349859 and rs13202464, respectively, lying in close proximity to non-coding sequences around the Mhc-B region. As expected, neither the mum nor the son had inherited these SNPs (Supplementary Table 5).

To confirm that these non-coding SNPs were indeed not present to modulate the disease severity in all our AS monkeys, we cross-checked rs4349859 (corresponding loci: Macaca_fascicularis_5.0:4:139402704) by SNP shot sequencing and that returned no corresponding SNP change in any of the disease animal nor in the healthy control. This further confirms that the disease-relevant MHC Class I allele do not fall within the proximity of Mhc-B locus unlike the human paralog. In parallel, SNP shot sequencing on another non-synonymous coding SNPs (corresponding loci: Macaca_fascicularis_5.0: chr6:95192910 and chr1:1600952276) in the genome of 55 monkeys (AS=34, control=21) was also performed and indeed none of such disease-prone SNPs existed in their genome.

With that in mind, we further perform in silico read mapping for the highly polymorphic MHC locus. Given the complexity for sequence alignment in the polymorphic region dubbed with unidentified introns with no reference genomic scaffold available for this species, we turned to conduct multiple alignments to known MHC Class I cDNA sequence instead. All MHC region mappable reads from the two individual WGS library construct from the healthy mother and her diseased son DNA respectively where stringency for read mapping was set to a high threshold to require exact sequence identity.

When aligned to our derived functional homolog sequences, we determined a roughly similar allele coverage for Mafa-A1*027 and Mafa-A2*007 in both the mother and her son, which suggested that these HLA alleles unlikely played a crucial role in the development of the disease. Nonetheless, the allele coverage scores in Mafa-A2*05 for the son were found to be approximately double that as in his biological mother, which strongly suggested that he manifested a diploid inheritance of Mafa-A2*05 as an HLA-B27 paralog from both parents. Furthermore, extended primer PCR had confirmed a higher copy number for this Mhc-A allele from DNA extracted from this mother-child pair, but not for the other two Mhc alleles or housekeeping gene loci.

Finally, we believe that recessive inherited patterns for protein-coding genes, especially those relevant to the innate immune response pathway may give rise to higher susceptibility to AS or other autoimmune diseases. Therefore, we also screened for missense recessive genetic traits that might turn out as useful predictors to the manifestation of the disease in macaques. In line with this endeavor, we performed filtering of minor allele SNPs with the following criteria: (1) missense coding mutations to that of the reference genome, where (2) the mother would maintain heterozygosity in the locus, and (3) the son shall inherit both recessive copies. A total of 38 such SNPs were found where only 15 amongst them are annotated genes.

A total of 13 known genes matching the above criteria were surveyed in 5 AS monkeys together with 5 control monkeys (Table 4). We found that within these sampled monkeys, most of them demonstrate either predominant nucleotide usage in DIEXF & ARSA (C only); CFB (T only), or random segregated nucleotide usage in SLC26A1 & ZBTB42 (C/G); SCGB3A1 & GNA15 (G/A); LILRB4 (T/A) at the missense loci; therefore, there is no enrichment for a particular SNP in these genes regardless of the disease phenotype.

**Table 4:** 38 SNP list

Chromosome	Position	Gene	Type	Reference	Son	Mum
1	1215288918	LOC102136658	Missense variant	C	T	*,T
1	168420059	DIEXF	Missense variant	A	C	C,T
3	1175485315	LOC102131469	Missense variant	A	C	C,T
3	1175880743	LOC102131469	Missense variant	T	A	A,G
3	1177214755	LOC102128920	Missense variant	A	C	*,C
3	1192009442	VIPR2	Missense variant	C	G	G,T
3	128778926	LOC102131469	Missense variant	T	G	TTTG,G
3	129229344	LOC102131469	Missense variant	A	G	*,G
4	111102645	SOD2	Missense variant	A	G	C,G
4	1138903304	CFB	Missense variant	G	T	*,T
4	1141429218	LOC102129714	Missense variant	C	T	A,T
4	1168015542	LOC107129646	Missense variant	A	T	G,T
5	165508494	LOC102126139	Missense variant	T	G	A,G
5	1972800	SLC26A1	Missense variant	C	T	G,T
6	1135164413	RUBCN	Missense variant	A	C	*,C
6	1180619461	SCGB3A1	Missense variant	C	G	*,G
7	1135135117	LOC102118143	Missense variant	C	G	G,T
7	1135882110	LOC102127558	Missense variant	C	G	G,A
7	1170188487	ZBTB42	Missense variant	C	G	G,*
7	156240664	LOC102126566	Missense variant	C	T	*,T
7	157176892	LOC102147189	Missense variant	C	A	A,T
8	122246303	NUDT18	Missense variant	A	G	C,G
9	139668835	LOC102143735	Missense variant	T	A	G,A
9	1708235	TUBB8	Missense variant	T	G	C,G
10	1197740	ARSA	Missense variant	T	C	C,G
10	178143092	LOC102125072	Missense variant	C	T	T,*
11	111981753	LOC107126647	Missense variant	G	T	*,T
13	115107917	LOC107127060	Missense variant	T	G	A,G
14	1120156195	LOC102147067	Missense variant	T	G	*,G
19	112305239	ZNE433	Missense variant	T	A	A,G
19	13139328	GNA15	Missense variant	T	G	A,G
19	13710621	LOC102134537	Missense variant	T	G	G,*
19	140352252	FCGBP	Missense variant	C	A	G
19	155499885	LOC102143631	Missense variant	C	T	T,A
19	155567454	LILRB4	Missense variant	C	T	A,T
19	155612771	LOC102144021	Missense variant	C	G	G,T
Scaffold 2511	1344	LOC102114865	Missense variant	C	G	G,A
Scaffold 26	1176274	LOC107128638	Missense variant	T	G	C,G

However, in most sampled AS animals (4/5), the substitution of G in the VIPR2 gene shall produce arginine residues instead when the predominant genotype of homozygous T shall encode serine residues in non-disease controls at the same locus. Likewise, we observed a tendency for the presence of A (2/5) in place of G which was the predominant SNP for control animals at the locus in ZNF433. In this locus, the presence of A rendered replacement of arginine to serine residues.

## Discussion

Epidemiological studies have found that the incidence of AS patients in China is about 0.2–0.54% [2]. Although the incidence rates are not fully consistent across countries, they are mainly between 0.3 and 0.5% [1]. Among the 20,000 cynomolgus monkey populations in the farm, 57 were diagnosed as suffering from AS; the spontaneous incidence rate of AS in monkeys was in line with that of clinical cases at about 0.28%. In addition, the conversion relationship between human and monkey age (1-year-old cynomolgus monkey is about 4 years old in human beings) provided a rough estimate for the onset age of AS animals also similar to human epidemiology, running along their families. However, our statistical analysis failed to re-establish the gender disparity in these monkeys, but this could be a biased statistic with a preference for female housing on the farm since male animals were frequently sold off. Therefore, we believe the current odds ratio overestimated the incidence in the female with confounding bias in terms of gender susceptibility.

Radiographic examination forms the basis for the clinical diagnosis of AS. Sacroiliitic joint lesion is a hallmark of AS, especially prominent in earlier disease stages, followed by multiple manifestations such as the spinal and peripheral joint lesions [1]. The lesions of the vertebral body are the most distinctive, with overt pathological changes consisting of bone erosion and syndesmophyte formation. CT scan showed that the sacroiliitic joint surface of the AS cynomolgus monkey had a rough joint surface, narrow joint space that diminished in the final stage. X-ray examination demonstrated bone erosion manifests as a square vertebra where the corner would erode, followed by syndesmophyte growth with adjacent vertebral bodies fusing together resulting in the typical “Bamboo-like” spine. The above process was found to match perfectly to clinical features as in AS patients along our 2-year follow-up in these diseased monkeys.

After multiple X-ray examinations over the 2 years, we found that the majority of bone deterioration occurred within the first half-year where the vertebral joints underwent transformation, starting from bone erosion to syndesmophyte formation at an accelerated pace when compared to humans. Nonetheless, the change from initial syndesmophyte formation to the final bamboo-like change requires a relatively longer time. The gradual deterioration at a shorter window when compared to humans is conducive to better evaluation for drug efficacy and pharmacology.

Clinically, it still lacks a gold standard for the diagnosis of AS. It is indeed difficult to distinguish AS from other similar diseases like rheumatoid arthritis (RA) only by radiographic examination during early stages [6, 24]. With a wider scope of research on AS in recent years, specific detection markers were identified for AS, including CRP [25], RF [26], IL-17 [15, 16], TNF-α [19], etc. A combined X-ray imaging and marker changes of CRP, RF, IL-17, and TNF-α detection provide a better comprehensive diagnostic account for diseased animals, where we confirm the existence of a spontaneous AS cynomolgus monkey model.

The hematological examination is usually not regarded as a key indicator of clinical diagnosis of AS mainly due to non-specific hematological changes related to systemic inflammation. However, these remain consistent features reflective of conditions in AS patients [25, 27–29]. In addition, the percentage of lymphocytes decreased significantly, and the percentage of neutrophils increased significantly also consistent as in AS patients [25], where neutrophils were defined to play a part in AS pathogenesis. Finally, the serum levels of TNF-α, IL-6, IL-4, and VEGF in AS cynomolgus monkeys are elevated, which are similarly consistent as in clinical research [17, 18]. Correspondingly, the significant drop in serum calcium and phosphorus levels might indicate the involvement of these minerals in the calcification of the ligaments at the lesions.

Based on the radiographic examination, we further analyzed pathological changes in AS cynomolgus monkeys. Heterotopic ossification of the intervertebral disc cartilage was found in anatomical specimens with no obvious abnormalities in the radiographic examination, indicating that the pathological examination revealed in vivo subtle lesions earlier but accurately. Several features, including synovitis, cartilage ossification, bone formation, and chondroid metaplasia, as well as cartilage destruction, are commonly found in the joints of AS patients [7]. In early lesions, synovial thickening, inflammatory cell infiltration, fibrous tissue hyperplasia, vascular proliferation, and cartilage destruction were found in the anterior ligament of the animal vertebral body. In the late stage, the vertebral body was more common in the formation of spurs, and the chondroid metaplasia foci were visible in the spur area. There was no obvious inflammatory cell infiltration, while the site was laden with active osteoblasts and osteoclasts. These observations have completely reproduced the pathological changes as observed in clinical AS patient specimens [8, 30] and have further reinstated the diagnosis of spontaneous AS in these animals.

More importantly, we gathered new evidence in establishing controversial issues raised in prior animal research through careful observation of the whole vertebral body section in these monkeys. The issue is about the presence of vascularized fibrous tissue in the diseased joint, an outgrowth also known as the granulation tissue, with an uncertain origin [7]. They are suggested to originate from the synovium or bone marrow, however lack of clear evidence to support such a claim.

After analyzing the human biopsy specimen, Bleid concluded that the fibrous tissue was from the bone marrow; firstly eroding the subchondral plate and then into the cartilage surface [8]. We argue against this prior finding but suggest that these fibrous structures appear more frequently between the synovial membrane and the disc annulus fibrosus, particularly at the edges where there are aggregated osteoclasts in the fibrous tissue as considered necessary for degradation of the cartilage matrix. Summing up, it is more likely that these vascularized fibrous tissues are more likely to be derived from the synovium.

In fact, we report the first observation of this phenomena since it had neither been encountered in mice with progressive ankylosis [31], in HLA–B27 transgenic rats with experimental inflammatory disease [32, 33], in HLA–B27 transgenic mice [10], nor in animals with murine ankylosing enthesopathy [34]. Intriguingly, it was even confirmed that no lesions of the spine were even present in the latter two models.

The earliest definition of AS as a hereditary disease was mainly due to the higher recurrence risk of AS than general non-hereditary disease, with a 63% recurrence risk in monozygotic twins [35] and a 8.2% recurrence risk in first-degree relatives [36]. Through genome-wide association studies, more and more genetic loci have been found to be associated with AS [2, 37, 38]. More than 100 genetic loci have been associated with AS, but they only summarized up to 30% of AS heritability [5]. Among those genes, HLA-B27, ERAP1, and IL-23R received much attention, accounting for the population-attributable risks at approximately 90%, 26%, and 1%, respectively [24, 28]. Interestingly, how these genotypes shall affect the pathogenesis of AS remains unknown.

A specialized genomic region in the short arm of human chromosome 6 encoded the polymorphic cell-membrane-bound glycoprotein MHC classizcal class I and class II molecules known as human leukocyte antigen (HLA) locus. The major function of this locus is to regulate immune response by the presentation of processed foreign peptides to circulating T lymphocytes. The polymorphism at the MHC region was accounted for the donor/recipient incompatibility in organ transplant rejection.

Despite their frequent use as a model in clinical research, a genetic study in cynomolgus is very limited especially in highly polymorphic regions such as the MHC locus. To complicate the matter, the whole macaque genus devised MHC polymorphism involving duplication of gene clusters with functional redundancy, unlike humans with functional exclusiveness in single HLA loci for each class. These disadvantages had limited clinical studies in all macaque models associated with graft-versus-host disease, infectious diseases as well as autoimmune-related diseases including AS, not to mention genotype-phenotype correlations and establishment of causal relationships. Nonetheless, macaques remained a good model to study human autoimmune diseases when they frequently demonstrated significant cross-reactivity of antibodies raised against human antigens.

HLA-B27 was singled out as an AS-susceptibility MHC allele for nearly half a decade. Interactors along the innate immunity signaling pathway ERAP1 and IL23R were also named as important contributors to AS-associated joint inflammation. Indeed, the IL-23/Th17 pathway influences the expression levels of genes involved in the differentiation of Th17/Th1 cells while the abnormal function of ERAP1 affected peptide processing which led to peptide–MHC-I complex misfolding. However, there is no well-defined B27 homolog in the Macaca genus, nor the characterization of its downstream pathway.

As confirmed from our healthy mother—diseased son WGS analysis backed by SNP shot sequencing in a wide screen, there were no minor alleles detected in SNP genotyping of these non-synonymous nor the HLA-B associated non-coding loci in AS or control animals. Likely, the Mafa-B regions do not directly contribute to the disease phenotypes within this group of AS monkeys.

With that, we would like to define our Mafa homolog of HLA-B27. When different macaque species were first compared, it was revealed more than 100 alleles with perfect match in MHC-B locus, which presumed that MHC-B polymorphism existed before the speciation of the macaques. Nonetheless, Mamu-A and Mafa-A sequences were found to be interspersed along the phylogenetic tree, while the vast majority of the alleles are unique in their species [39]. Furthermore, Mamu-B genes were already reported to demonstrate a closer relationship with HLA-C than with HLA-B. Therefore, the locus distribution of MHC I molecules does not always conform to sequence consistency and functionality along primate evolution.

In fact, based on the peptide-binding specificity of Mamu-A2*01, Mamu-A2*05, *Mamu-*B0*03 Mamu-B0*08, and *Mamu-*B0*10 were all reported to be analogous to those alleles found in the HLA-B27 supertype family [20, 40]. This seemingly also indicates Mhc-A & Mhc-B may also include alleles homologous to the AS susceptibility locus. To facilitate the hunt in disease-associated MHC allele, we had performed read mapping of WGS reads back to a genome sequence repository of Mafa MHC cDNA and revealed significant mapping to Mhc-A genes, particularly rendered exact matches in Mafa-A1*027:02, Mafa-A1*007:04, and Mafa-A2*05:48, which appeared as top hits when conducting protein blast compared to HLA-B2705. The doubled coverage in diseased son compared to its healthy mother strengthened our beliefs in the usage of this HLA allele in place of HLA-B27 in Cynomolgus.

Finally, homozygous recessive inheritance of missense coding genes may undermine the onset of AS in the population as a whole. After screening the SNP genotypes in healthy and AS monkeys in gender-mixed groups of 5, it was revealed that most healthy macaques carried a homozygous T in the VIPR2 gene where the disease ones carried predominantly homozygous G instead. In fact, it was previously revealed in mice encoding a genetic deletion of this gene shall exacerbate the autoimmune disease EAE by increased production of proinflammatory cytokines such as TNF-α, IL-6, and IL-17 in the CNS and lymph nodes [41]. Moreover, the presence of A instead of the predominant G at our surveillance site also led to a missense mutation in replacing arginine (positively-charged) with serine (neutral) residues. Given that zinc finger proteins such as ZNF433 we report here are transcription factors, any changes in total charge may affect factor binding to negatively charged DNA. This gene had also been implicated as a disease-susceptible locus in multiple sclerosis, which is also an IL-23/Th17-related autoimmune disorder [42, 43].

## Conclusions

In summary, there is a lack of relevant research models to deepen our understanding of the etiology of AS. Indeed, the heavy reliance on individual genetic factors or inflammatory effects only severely hampered AS research till now. Furthermore, the scarcity of sample collection and frequent ethical concerns further hindered a probable comprehensive study to be performed in real AS patients. Our current study evaluated AS cynomolgus monkeys from multiple aspects such as epidemiology, hematology, radiographic imaging, pathology, and genetic linkage analysis, all demonstrating strikingly similar features to that in clinical AS patients. We therefore advocate our spontaneous disease Cynomolgus monkey as an excellent surrogate model to pave groundbreaking discovery of AS etiology and drug efficacy.

## Supplementary Information

**Additional file 1: Supplementary Table 1.** Information for ELISA kit.

**Additional file 2: Supplementary Table 2.** Sequences of primers used in SNP genotyping.

**Additional file 3: Supplementary Table 3.** Curvatures of joints (mean±sd).

**Additional file 4: Supplementary Table 4.** Stretches of joints (mean±sd).

**Additional file 5: Supplementary Table 5.** Consistency between *Homo sapiens* and *Macaca fascicularis* for SNPs within previously confirmed risk loci.

## Data Availability

The datasets used and/or analyzed during the current study are available from the corresponding author on reasonable request.

## Abbreviations

ALB: Albumin
ALP: Alkaline phosphatase
ANOVA: Analysis of variance
Anti-CCP antibody: Anti-cyclic peptide containing citrulline antibody
AS: Ankylosing spondylitis
ASO: Anti-Streptolysin O
BM: Bone marrow
Ca: Calcium
CRP: C-reactive protein
CT: Computed tomography
ESR: Erythrocyte sedimentation rate
FC: Fibrocartilage
GLOB: Globulin
HB: Heterotopic bone
GT: Granulation tissue
H&E: Haematoxylin and eosin
HGB: Hemoglobin
IgM-RF: Immunoglobulin M-rheumatoid factor
IL: Interleukin
LYMPH: Lymphocyte
MCHC: Mean corpuscular hemoglobin concentration
mSASSS: Modified Stoke Ankylosing Spondylitis Spinal Score
MRI: Magnetic resonance imaging
NEUT: Neutrophils
PCT: Procalcitonin
PLT: Blood platelet
RA: Rheumatoid arthritis
ROM: Range of motion
SD: Standard deviation
SNP: Single-nucleotide polymorphism
TNF-α: Tumor necrosis factor α
TP: Total protein
VEGF: Vascular endothelial growth factor
WBC: White blood cell
