# Supplementary material for: A new and spontaneous animal model for ankylosing spondylitis is found in cynomolgus monkeys
Source: Arthritis Res Ther. 2022 Jan 3;24:1. doi: 10.1186/s13075-021-02679-5 (PMC8722021; doi:10.1186/s13075-021-02679-5)
Supplement: Supplementary file 1 — Additional file 1: Supplementary Table 1. Information for ELISA kit. [file 13075_2021_2679_MOESM1_ESM.docx]

**Supplementary Table. 1** Information for ELISA kit

| **Reagent Name** | **Manufacturer** | ****Item NO.**** |
| --- | --- | --- |
| Monkey C-reactive protein (CRP) ELISA kit | SHANGHAI BANGYI BIOTECHNOLOGY CO. , LTD. | BYE90039 |
| Monkey Anti-cyclic peptide containing citrulline antibody(CCPAb) ELISA kit | SHANGHAI BANGYI BIOTECHNOLOGY CO. , LTD. | BYE90071 |
| Monkey Anti-Streptolysin O (ASO) ELISA kit | SHANGHAI BANGYI BIOTECHNOLOGY CO. , LTD. | BYE90357 |
| Monkey Procalcitonin (PCT) ELISA kit | SHANGHAI BANGYI BIOTECHNOLOGY CO. , LTD. | BYE90225 |
| Monkey lgG rheumatoid factors(lgG-RF) ELISA kit | SHANGHAI BANGYI BIOTECHNOLOGY CO. , LTD. | BYE90058 |
| Monkey lgM rheumatoid factors(lgM-RF) ELISA kit | SHANGHAI BANGYI BIOTECHNOLOGY CO. , LTD. | BYE90062 |
| NHP Custom ProcartaPlex 9-plex | Invitrogen | 197642000 |
