# Supplementary material for: A new and spontaneous animal model for ankylosing spondylitis is found in cynomolgus monkeys
Source: Arthritis Res Ther. 2022 Jan 3;24:1. doi: 10.1186/s13075-021-02679-5 (PMC8722021; doi:10.1186/s13075-021-02679-5)
Supplement: Supplementary file 2 — Additional file 2: Supplementary Table 2. Sequences of primers used in SNP genotyping. [file 13075_2021_2679_MOESM2_ESM.docx]

**Supplementary Table. 2** Sequences of primers uesd in SNP Genotyping

| **Primers** | **Sequence** | **Length(bp)** | **Extension primer** |
| --- | --- | --- | --- |
| ERAP1 | **Sense:** 5’- GGGCTGGATGTGAAAACCAT -3’  **Antisense:** 5’- ATCACAGTGAGGGGAAGGAAT -3’ | 84 | TGATGAACACTTGGACACTGCAGA |
| IL-23R | **Sense:** 5’- ACGTGAGAATTCCA GGAGC -3’  **Antisense:** 5’- AAGCATGTTCCACCTTCAGA -3’ | 96 | CTGACTGACTGATAAATTTTAGCCATTCTTCTGCCT |
| MHC-B | **Sense:** 5’- CATGCTCTTTAGATAAACTCCCTT -3’  **Antisense:** 5’- TGAAAGCAGCCTAAT CCTCTTA -3’ | 84 | CTGACTGACTGACTGACTGACTTACATGTCTTTGTACCTACT |
