# Supplementary material for: A new and spontaneous animal model for ankylosing spondylitis is found in cynomolgus monkeys
Source: Arthritis Res Ther. 2022 Jan 3;24:1. doi: 10.1186/s13075-021-02679-5 (PMC8722021; doi:10.1186/s13075-021-02679-5)
Supplement: Supplementary file 3 — Additional file 3: Supplementary Table 3. Curvatures of joints (mean±sd). [file 13075_2021_2679_MOESM3_ESM.docx]

**Supplementary Table. 3** Curvatures of joints(mean±sd)

| Joints | Control | AS |
| --- | --- | --- |
| Right hip joint | 27.90±6.703 | 32.43±7.061 |
| Left hip joint | 26.25±7.063 | 29.50±7.912 |
| Right knee joint | 34.05±8.003 | 23.02±5.120 |
| Left knee joint | 31.75±7.873 | 23.88±5.370 |
| Right elbow joint | 29.70±7.706 | 28.67±7.077 |
| Left elbow joint | 30.80±6.170 | 26.10±7.244 |
| Spine | 0.35±1.565 | 20.33±18.86 |
