# Supplementary material for: A new and spontaneous animal model for ankylosing spondylitis is found in cynomolgus monkeys
Source: Arthritis Res Ther. 2022 Jan 3;24:1. doi: 10.1186/s13075-021-02679-5 (PMC8722021; doi:10.1186/s13075-021-02679-5)
Supplement: Supplementary file 4 — Additional file 4: Supplementary Table 4. Stretches of joints (mean±sd). [file 13075_2021_2679_MOESM4_ESM.docx]

**Supplementary Table. 4** Stretches of joints(mean±sd)

| Joints | Control | AS |
| --- | --- | --- |
| Right hip joint | 162.8±9.808 | 137.3±19.50 |
| Left hip joint | 161.0±12.56 | 133.4±22.54 |
| Right knee joint | 24.45±11.59 | 95.24±36.19 |
| Left knee joint | 12.30±11.73 | 92.17±30.46 |
| Right elbow joint | 15.35±8.242 | 16.43±11.75 |
| Left elbow joint | 24.15±9.896 | 20.14±15.11 |
