# Supplementary material for: A new and spontaneous animal model for ankylosing spondylitis is found in cynomolgus monkeys
Source: Arthritis Res Ther. 2022 Jan 3;24:1. doi: 10.1186/s13075-021-02679-5 (PMC8722021; doi:10.1186/s13075-021-02679-5)
Supplement: Supplementary file 5 — Additional file 5: Supplementary Table 5. Consistency between Homo sapiens and Macaca fascicularis for SNPs within previously confirmed risk loci. [file 13075_2021_2679_MOESM5_ESM.docx]

**Supplementary Table. 5** Consistency between Homo sapiens and Macaca fascicularis for SNPs within previously confirmed risk loci

|  | Gene:Consequence | Ref(GRCh38) | Ref(Maca fascicularis 5.0) | Genomic Location (GRCh38.p12) | Cenomic Loation (Macaca fascicularis 5.0)\| | Mum | Son |
| --- | --- | --- | --- | --- | --- | --- | --- |
| rs27434 | ERAP1:Synonymous Variant | A | NA | chr5:96793809 | NA |  |  |
| rs30187 | ERAPI:Missense Variant | T | T | chr5:96788627 | NC 02227.1:95192910 | T | T |
| rs11209026 | 1L23R:Missense Variant | G | NA | chr1:67240275 | NA |  |  |
| rs1004819 | IL23R:lntron Variant | G | NA | chr1:67204530 | NA |  |  |
| rs10489629 | IL23R:lntron Variant | T | NA | chr1:67222666 | NA |  |  |
| rs11465804 | 1L23R:lntron Variant | T | T | chr1:67236843 | NC 022272.1:160116074 | NA | NA |
| rs1343151 | IL23R:lntron Variant | G | NA | chr1:67253446 | NA |  |  |
| rs10889677 | IL23R:3 Prime UTR Variant | C | C | chr1:67259437 | NC 022272.1:160095226 | NA | NA |
| rs11209032 | None | G | NA | chr1:67274409 | NA |  |  |
| rs1495965 | None | C | C | chr1:67287825 | NC 022272.1:160066841 | NA | NA |
| rs4349859 | MICA-ASI:Intron Variant,MICA:2KB Upstream Variant | G | NA | chr6:31398010 | NA |  |  |
| rs13202464 | None | A | NA | chr6:31376806 | NA |  |  |
| rs4389526 | ANTXR2:Intron Variant | T | NA | chr4: 80025321 | NA |  |  |
| ra6556416 | None | A | NA | chr5:159391737 | NA |  |  |
| rs2297909 | KIF21B:lntron Variant | G | NA | chrl:200991179 | NA |  |  |
| rs11249215 | None | G | G | chr1:24970693 | NC 022272.1:203384526 | NA | NA |
| rs11616188 | None | G | NA | chr12:6393576 | NA |  |  |
| rs8070463 | None | T | T | chr17:47691470 | NC 022287.1:33007498 | T | T |
| rs10440635 | None | G | NA | chr5:40490688 | NA |  |  |
| rs10781500 | CARD9:2KB Upstream Variant | C | NA | chr9:136374886 | NA |  |  |
